# Synthetic biology increases efficiency of *Escherichia coli *to produce *Parawixia bistriata *spider silk protein

**DOI:** 10.1186/1753-6561-8-S4-P231

**Published:** 2014-10-01

**Authors:** Valquíria Alice Michalczechen-Lacerda, Olena Tokareva, Andressa de Rezende Bastos, Marina Silveira da Silva, Giovanni Rodrigues Vianna, André Melro Murad, David Lee Kaplan, Elíbio Leopoldo Rech

**Affiliations:** 1 Embrapa Genetic Resources and Biotechnology-Parque Estação Biológica, Brasilia, DF, 70770-917, Brazil; 2 University of Brasilia,Campus Darcy Ribeiro, Brasilia, DF, 70910-900, Brazil; 3 Tufts University, Medford, MA, 02155, USA

## Background

Spider dragline silk is considered to be the toughest biopolymer on Earth due to an extraordinary combination of strength and elasticity. With synthetic biology it is possible to express recombinant spider silk proteins, which are characterized by a highly repetitive rich glycine and alanine sequence [1]. However, production of high molecular weight spider silk protein can be difficult due to DNA instability, transcription and translation errors. Here we show, for the first time, Masp2 (105 kDa) spidroin silk protein production from the Brazilian spider *Parawixia bistriata *in different metabolically engineered *E.coli *strains.

## Methods

A *Masp2 *monomer gene from *P. bistriata *was designed (DNA2.0), and a 32 mer plasmid was constructed [2]. To increase the glycyl-tRNA pool, tRNA^Gly ^and glycyl-tRNA synthetase genes were cloned in pACYC184 plasmid [3]. All vectors were confirmed by DNA sequencing. The bacterium BL21(DE03) was co-tranformed with the 32 mer and one metabolic plasmid. pACYC184 and pET19b vectors was used as controls. Cells were grown in a 2L flask culture with 1L of LB medium with antibiotics, at 37°C and 200 rpm. Silk proteins were induced with 1 mM IPTG, for four hours, and growth curves were established. Protein histag N-terminal extraction was performed under native conditions and purified by IMAC. All samples were analyzed by SDS-PAGE gels, staining with Colloidal Blue and by Western blot. Dialysis was at 4°C against 10 mM Tris-HCl pH 8.0 for the first 24 hours, and water for an additional 24 hours. Samples were lyophilized and weighed. Statistical analyses were determined by ANOVA and unpaired Tukey test (ASSISTAT 7.7 BETA 2013), bein statistically significant at *P*<0.01.

## Results and conclusions

All *E. coli *that received Masp2 grew, expressing spider protein, and data yields are shown in table 1 (N = 3). There were no expression differences between BL21(DE03) and pACYC184, nor in the strain containing one gene copy to increase tRNA^Gly ^pool. This shows no interference from the initial plasmid used for bacterial metabolic engineering. The amount of tRNA was probably not enough to supply the metabolic stress. When the tRNA^Gly ^and glycine pool were overexpressed by ptetgly2, pTetglyVXY-glyA and pTetgly2-glyA, an improvement was noted in the production of 105 kDa Masp2 (P <0.01). In similar studies, the same synergistic effect was reported for high molecular weight protein from *Nephila clavipes *[3]. The same authors also reported that protein sizes are directly associated with fiber quality properties. The 105 kDa Masp2 produced was able to be spun in fibers, which will be characterized in future analyses. Currently, there is no ideal heterologous organism to produce spider silk proteins, and metabolic engineering together with synthetic biology can optimize spider silk protein production. Different spider silk proteins may result in new types of protein-based biomaterials with wide applications in medicine and industry.

**Table 1:** Spider silk Masp2 protein yield in different engineered *E.coli*

Bacteria	Means	*
pTetgly2-glyA	3.39	a
pTetglyVXY-glyA	2.83	ab
pTetgly2	2.40	b
pTetglyVXY	0.96	c
pACYC184	0.99	c
BL21(DE03)	0.79	c

Means reference: mg protein/g pellet. *P *< 0.01; CV = 13.72%

## Acknowledgements

Embrapa Genetics Resources and Biotechnology, University of Brasilia, CNPq, CAPES, FAPDF, the NIH P41 Resource Center (EB002520), Tufts University.
